# Expert consensus on the monitoring and treatment of sepsis-induced immunosuppression

**DOI:** 10.1186/s40779-022-00430-y

**Published:** 2022-12-26

**Authors:** Fei Pei, Ren-Qi Yao, Chao Ren, Soheyl Bahrami, Timothy R. Billiar, Irshad H. Chaudry, De-Chang Chen, Xu-Lin Chen, Na Cui, Xiang-Ming Fang, Yan Kang, Wei-Qin Li, Wen-Xiong Li, Hua-Ping Liang, Hong-Yuan Lin, Ke-Xuan Liu, Ben Lu, Zhong-Qiu Lu, Marc Maegele, Tian-Qing Peng, You Shang, Lei Su, Bing-Wei Sun, Chang-Song Wang, Jian Wang, Jiang-Huai Wang, Ping Wang, Jian-Feng Xie, Li-Xin Xie, Li-Na Zhang, Basilia Zingarelli, Xiang-Dong Guan, Jian-Feng Wu, Yong-Ming Yao, the Shock and Sepsis Society of Chinese Research Hospital Association, the China Critical Care Immunotherapy Research Group, the International Federation of the Shock Societies (IFSS)

**Affiliations:** 1 Department of Critical Care Medicine, the First Affiliated Hospital of Sun Yat-Sen University, 58 Zhongshan Er Road, Yuexiu District, Guangzhou, 510080 Guangdong China; 2 Translational Medicine Research Center, Medical Innovation Research Division and Fourth Medical Center of the Chinese PLA General Hospital, 28 Fuxing Road, Haidian District, Beijing, 100853 China; 3 Department of Burn Surgery, the First Affiliated Hospital of Naval Medical University, Shanghai, 200433 China; 4 Ludwig-Boltzmann Institute for Experimental and Clinical Traumatology, 1200 Vienna, Austria; 5 Department of Surgery, University of Pittsburgh Medical Center, Pittsburgh, PA 15213 USA; 6 Center for Surgical Research and Department of Surgery, University of Alabama at Birmingham, Birmingham, AL 35294 USA; 7 Department of Critical Care Medicine, Ruijin Hospital, Shanghai Jiaotong University, Shanghai, 200025 China; 8 Department of Burns, the First Affiliated Hospital of Anhui Medical University, Hefei, 230022 China; 9 Department of Critical Care Medicine, Peking Union Medical College Hospital, Chinese Academy of Medical Science and Peking Union Medical College, Beijing, 100730 China; 10 Department of Anesthesiology, the First Affiliated Hospital, Zhejiang University School of Medicine, Hangzhou, 31003 China; 11 Department of Critical Care Medicine, West China Hospital of Sichuan University, Chengdu, 610041 China; 12 Department of Critical Care Medicine, General Hospital of Eastern Theater Command of Chinese PLA, Nanjing, 210002 China; 13 Department of Surgical Intensive Critical Unit, Beijing Chao-Yang Hospital, Capital Medical University, Beijing, 100020 China; 14 State Key Laboratory of Trauma, Burns and Combined Injury, Research Institute of Surgery, Daping Hospital, Army Medical University, Chongqing, 400042 China; 15 Department of Critical Care Medicine, Fourth Medical Center of the Chinese PLA General Hospital, Beijing, 100048 China; 16 Department of Anesthesiology, Nanfang Hospital, Southern Medical University, Guangzhou, 510515 China; 17 Department of Critical Care Medicine and Hematology, the Third Xiangya Hospital, Central South University, Changsha, 410000 China; 18 Emergency Department, the First Affiliated Hospital of Wenzhou Medical University, Wenzhou, 325000 China; 19 Department of Traumatology and Orthopedic Surgery, University Witten-Herdecke, 51109 Cologne, Germany; 20 Critical Illness Research, Lawson Health Research Institute, London Health Sciences Centre, London, ON N6A 4G4 Canada; 21 Department of Critical Care Medicine, Tongji Medical College, Huazhong University of Science and Technology, Wuhan, 430022 China; 22 Department of Intensive Care Unit, General Hospital of Southern Theater Command of Chinese PLA, Guangzhou, 510030 China; 23 Department of Burns and Plastic Surgery, Affiliated Suzhou Hospital of Nanjing Medical University, Suzhou, 215002 China; 24 Department of Critical Care Medicine, Harbin Medical University Cancer Hospital, Harbin, 150081 China; 25 Children’s Hospital of Soochow University, Pediatric Research Institute of Soochow University, Suzhou, 215123 China; 26 Department of Academic Surgery, University College Cork, Cork University Hospital, Cork, T12 E8YV Ireland; 27 Center for Immunology and Inflammation, the Feinstein Institute for Medical Research, Northwell Health, Manhasset, NY 11030 USA; 28 Department of Critical Care Medicine, Zhongda Hospital, School of Medicine, Southeast University, Nanjing, 210009 China; 29 Department of Pulmonary and Critical Care Medicine, Chinese PLA General Hospital, Beijing, 100853 China; 30 Department of Critical Care Medicine, Xiangya Hospital, Central South University, Changsha, 410008 China; 31 Division of Critical Care Medicine, Cincinnati Children’s Hospital Medical Center, Cincinnati, OH 41073 USA; 32 Guangdong Clinical Research Center for Critical Care Medicine, Guangzhou, 510080 China

**Keywords:** Sepsis, Immune function monitoring, Immunomodulatory therapy, Immunosuppression

## Abstract

Emerged evidence has indicated that immunosuppression is involved in the occurrence and development of sepsis. To provide clinical practice recommendations on the immune function in sepsis, an expert consensus focusing on the monitoring and treatment of sepsis-induced immunosuppression was developed. Literature related to the immune monitoring and treatment of sepsis were retrieved from PubMed, Web of Science, and Chinese National Knowledge Infrastructure to design items and expert opinions were collected through an online questionnaire. Then, the Delphi method was used to form consensus opinions, and RAND appropriateness method was developed to provide consistency evaluation and recommendation levels for consensus opinions. This consensus achieved satisfactory results through two rounds of questionnaire survey, with 2 statements rated as perfect consistency, 13 as very good consistency, and 9 as good consistency. After summarizing the results, a total of 14 strong recommended opinions, 8 weak recommended opinions and 2 non-recommended opinions were produced. Finally, a face-to-face discussion of the consensus opinions was performed through an online meeting, and all judges unanimously agreed on the content of this consensus. In summary, this expert consensus provides a preliminary guidance for the monitoring and treatment of immunosuppression in patients with sepsis.

## Introduction

Sepsis refers to life-threatening organ dysfunction caused by a dysregulated host response to infection [1]. According to the latest data on global disease burden, nearly 50 million individuals had sepsis worldwide in 2017, which resulted in 11 million deaths [2]. Indeed, sepsis has become one of the main global health challenges, imposing a huge burden on the society and economy in various countries. Emerged evidence indicates that immune dysfunction is involved in the occurrence and development of sepsis, and patients may present with both hyperinflammatory response and immunosuppression. The former initiates early tissue damage and organ dysfunction, while the latter, when severe and persistent, further induces a variety of lethal complications, which significantly increase mortality of patients in the mid and late stages of sepsis [3–5]. However, multiple clinical trials assessing anti-inflammatory strategies have not yielded promising results. In recent years, increasing attention has been paid to the tools that may effectively dismantle the immunosuppressive state of patients with sepsis [6].

Dynamic and accurate assessment of immune status is a prerequisite for timely identification of immune dysfunction in patients with sepsis and determining the timing of immunomodulatory therapy. However, due to insufficient understanding of the precise molecular mechanisms and cellular bases in sepsis-induced immunosuppression, clinical monitoring indicators and evaluation systems that can effectively reflect the immune status of patients with sepsis are lacking [7, 8]. Currently, lymphocyte count and monocyte human leukocyte antigen DR (mHLA-DR) are widely used to evaluate the immune function changes in septic patients, but still have some limitations. Although lymphocyte count is easily obtained, its specificity is relatively low due to many confounding factors; meanwhile, mHLA-DR detection requires complex equipment with high detection cost, and the definition of early warning threshold remains inconclusive [9]. In recent years, investigators have applied multi-omic approaches to explore immune monitoring, aiming to discover new sepsis-related cell subsets and biomarkers, yet their translational significance and value need to be further validated by large-scale clinical trials [10–12]. Meanwhile, researchers are still debating on patient selection, treatment timing, dosage, drug combination, treatment duration, indications for discontinuation, and efficacy evaluation for immunomodulatory therapy in patients with sepsis that has not been evaluated in large-scale, high-quality clinical randomized controlled trials (RCTs) [13–15]. Thus, a variety of factors make it difficult for clinicians to accurately evaluate and monitor immune status in patients with sepsis, and implementing effective immunomodulation as well as drug intervention in a targeted and timely manner remains unsatisfactory. Based on a large number of clinical findings, this expert consensus comprehensively surveyed two aspects of sepsis, including immune monitoring and immunotherapy, aiming to provide a reference for clinicians to understand the pathogenesis of sepsis-induced immunosuppression and effectively implement immune monitoring and immunomodulatory strategies.

## Methods

### Questionnaire design

**Fig. 1:**
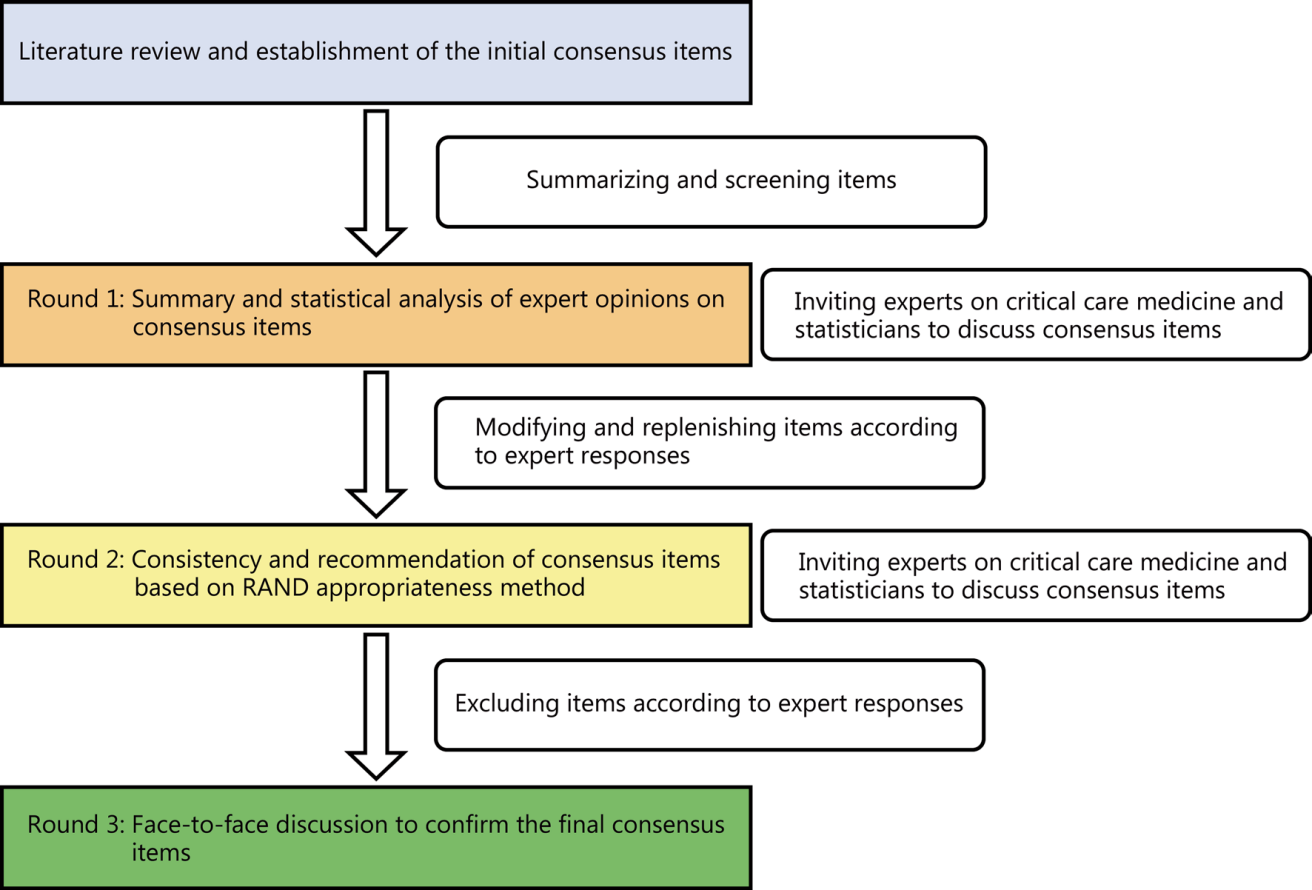
Flowchart Delphi process

A questionnaire design group was established, and retrieved and summarized publications related to sepsis immune monitoring and treatment by searching databases including PubMed, Web of Science (WOS), China National Knowledge Infrastructure (CNKI), etc.. Three rounds of group discussion were conducted, and the first version of the expert questionnaire was formulated (Fig. 1). There were 8 aspects with 27 items in the questionnaire survey, including whether immunosuppression exists in patients with sepsis, how to monitor immune function, high-risk factors for immunosuppression in patients with sepsis, commonly used monitoring indicators for sepsis-induced immunosuppression, how and when to initiate sepsis immunomodulatory therapy, characteristics of immunomodulatory drugs used, whether immunomodulatory therapy requires dynamic monitoring of immune function, and the endpoints of immunomodulatory therapy.

### Expert selection

Experts with extensive experience in sepsis immunomodulatory therapy were selected for consultation. Following the principles of authority and representativeness, a total of 22 experts were screened, and the consensus items were scored using an online questionnaire.

### Questionnaire content

The content of this questionnaire included the basic information of the experts, their recognition scores for all listed items, familiarity degree for all aspects, judgment basis of scores and degrees of influence. Meanwhile, to facilitate the experts to provide their feedback for potential revision of the items, the questionnaire included supplementary materials for expert suggestions.

The item recognition score used a 9-point scale, and each indicator had a total of 9 levels from “extremely inappropriate” to “very appropriate”, which were recorded from 1 to 9 in sequence. The degree of expert’s familiarity with each aspect was graded to 6 levels, which included (from high to low) 1.0, 0.8, 0.6, 0.4, 0.2 and 0 points. Among the four judgment bases (theoretical analysis, practical experience, reference to domestic and foreign materials, and intuition) those experts scored each aspect; each judgment basis had three situations: large, middle, and little. Table 1 shows the scores of impacts for each judgment basis.

**Table 1:** Impact degree scores of each judgment basis

Judgment bases	Impact degree scores of expert judgments		
	Large	Middle	Little
Theoretical analysis	0.3	0.2	0.1
Practical experience	0.5	0.4	0.3
Reference to domestic and foreign counterparts	0.1	0.1	0.1
Intuition	0.1	0.1	0.1

### The RAND appropriateness method (RAM)

#### Screening method for questionnaire items

Based on the RAM, the results of the expert consultation were sorted, and meanwhile the appropriateness of each item and the consistency of expert recommendation were summarized. These two parameters were used together to determine the recommendation for each item, i.e., whether the item was to be retained or modified based on the RAM result.

#### Item appropriateness

The expert judgement for each item was graded to 9 sequential levels, with 9 being “very appropriate” and 1 being “extremely inappropriate”. In addition, the 9 scoring levels were divided into 3 intervals, including 7–9 classified as “appropriate area”, 4–6 classified as “uncertain area”, and 1–3 classified as “inappropriate”.

#### Consistency of item scoring

Item consistency was determined by whether > 70% of the entire population scored within the interquartile range (IQR), and the median value and the degree of dispersion around the median value were determined. Based on the above information, a consensus of expert opinion for each item could be derived (Table 2).

**Table 2:** Definition of consistency

Terms	Definition
Perfect consistency	All experts scored 7–9
Very good consistency	Expert scores for the median and IQR were the same integer or 80% of the expert scores were within one integer from the median value
Good consistency	50% of the expert scores were within one integer from the median value or 80% of the expert scores were within two integers from the median value
Some consistency	50% of expert scores were within two integers from the median value or 80% of the expert scores were within three integers from the median value
No consistency	All other cases. Any dissenting median value

*IQR* interquartile range

#### Item recommendation

Table 3 summarize the strength of recommendations for the items.

**Table 3:** Definition of recommendations

Terms	Definition
Strong recommendation	The following three conditions should be met: (1) A proportion of scores within the IQR greater or equal to 70% (2) A degree of consistency at least “very good” (3) A median score not in the uncertain area, i.e., 4–6
Weak recommendation	The following three conditions should be met: (1) A proportion of scores within the IQR greater or equal to 70% (2) A degree of consistency of “good or some” and any median score, or any degree of consistency and a median score of 4–6 (3) A median score not in the uncertain area, i.e., 4–6
No recommendation	One of the following three conditions should be met in this case: (1) A proportion of scores within the IQR below 70% (2) No consistency (3) A median score in the uncertain area (4–6) combined with any consistency

*IQR* interquartile range

### Statistical analysis

Statistical analysis was performed with R 4.1.1. The following indicators were calculated: 1) the positive coefficient of experts, which was the questionnaire recovery rate; 2) the expert judgment coefficient (Ca) for each item, which was the sum score of each expert’s judgment basis for each item, the familiarity coefficient (Cs) and authority coefficient (Cr) [Cr = (Ca + Cs)/2], with the results expressed as mean ± standard deviation; 3) mean, standard deviation, full score ratio and coefficient of variation for each item score. We then calculated the Kendall’s coordination coefficient for each category to assess whether the experts’ scoring of each item was consistent.

## Results

### Statistical results

#### Basic information of experts

A total of 22 targeted experts in the field of sepsis immunomodulatory therapy were selected, and all 22 questionnaires were returned. The median age of the experts was 54.5 (IQR 44.3–57.8) years, for a median working duration of 31.0 (IQR 21.5–35.0) years. The basic information of the experts is shown in Table 4.

**Table 4:** Basic information of the participating experts

Parameters	Number
Recovery rate	100%
Response rate	100%
Age (years)	
< 45	6
45–60	14
> 60	2
Sex	
Male	19
Female	3
Education	
Doctor of Medicine	21
Master	1
Professional title	
Senior professor	21
Vice-senior professor	1
Years of professional experience	
< 20	4
20–30	6
> 30	12
Department	
Intensive Care Unit (ICU)	12
Trauma/Burn Division/Department	4
Anesthesiology Department	2
Others (Emergency, Hematology, Respiratory, and Surgery Department)	4

#### The degree of enthusiasm in experts

A total of 22 questionnaires were distributed in this round, and all 22 were recovered with a recovery rate of 100%, indicating that the selected experts were highly motivated.

#### Concentration degree of experts’ opinions

The concentration degree of experts’ opinions was measured by calculating the mean, standard deviation, full score ratio (proportion of patients with full score for the item), coefficient of variation (standard deviation/mean value of the item’s scores) and Kendall’s coordination coefficient.

Except for immunomodulatory drugs, whose coefficient of variation for each item was large, the coefficients of variation for all other items were less than 0.25, indicating a high consistency. Meanwhile, immune monitoring, immunomodulatory therapy, and the Kendall’s coordination coefficient for each aspect were calculated separately. The Kendall’s coordination coefficients were 0–1, and the larger the value, the higher the consistency. The Kendall’s coordination coefficients of immune monitoring and immunomodulatory therapy were 0.28 and 0.32, respectively, both of which were statistically significant, with high consistency of expert scoring. Three out of eight aspects, including high risk factors of sepsis-induced immunosuppression, monitoring indicators of sepsis-induced immunosuppression and immunomodulatory drugs, had high Kendall’s coordination coefficients, while the other aspects had low Kendall’s coordination coefficients.

Based on a comprehensive consideration, the concentration of the scoring in the first round of the survey was satisfactory.

#### Expert authority

The Cr was calculated based on mean Ca and Cs. Generally, Cr ≥ 0.7 is defined as acceptable reliability. In this study, Cr in all aspects were greater than or equal to 0.8, indicating a high degree of expert authority in this study (Table 5).

**Table 5:** Concentration, coordination and authority of the consensus content

Consensus	Mean	Standard deviation	Full score ratio	Coefficient of variation	Coordination coefficient	Judgment coefficient	Familiarity coefficient	Authority coefficient
*Immune monitoring*					0.28^*^			
Patients with sepsis had immunosuppression					0.05	0.94 ± 0.10	0.72 ± 0.35	0.83 ± 0.20
Presence of innate immunosuppression	7.95	1.56	55	0.20				
Presence of acquired immunosuppression	8.23	1.23	59	0.15				
Sepsis-related immune function monitoring					0.09	0.95 ± 0.08	0.73 ± 0.35	0.84 ± 0.19
Initiated immune monitoring within 48 h after diagnosis of sepsis	7.95	1.40	50	0.18				
Need for dynamic monitoring of immune status	8.27	1.52	68	0.18				
Patients with sepsis had high risk factors for immunosuppression					0.49^*^	0.97 ± 0.06	0.76 ± 0.33	0.86 ± 0.18
Elderly (age ≥ 65 years old)	7.82	0.91	27	0.12				
Patients with malignant tumors under radiotherapy/chemotherapy within 3 months	8.41	0.73	55	0.09				
Patients under long-term immunosuppressive or steroids therapy	8.64	0.66	73	0.08				
Malnutrition (BMI < 18.5 kg/m^2^)	7.14	1.36	18	0.19				
Secondary infection	7.23	1.48	18	0.20				
Monitoring indicators of immunosuppression in patients with sepsis					0.24^*^	0.96 ± 0.07	0.73 ± 0.33	0.84 ± 0.18
Decreased mHLA-DR	8.18	0.85	41	0.10				
Reduced responsiveness of monocytes to endotoxin stimulation	7.68	1.04	23	0.14				
Decreased peripheral blood lymphocyte count	8.05	1.05	45	0.13				
Increased regulatory T cell ratio	7.41	0.85	9	0.12				
Th1/Th2 imbalance	7.45	1.22	27	0.16				
Decreased peripheral blood immunoglobulin (IgA, IgM and IgG) concentrations	6.55	1.54	9	0.23				
*Immunotherapy*					0.32^*^			
Immunomodulatory therapy for sepsis					0.02	0.92 ± 0.10	0.73 ± 0.31	0.82 ± 0.17
Percentage of mHLA-DR < 60%	7.05	1.29	9	0.18				
mHLA-DR < 15,000 AB/C	7.00	1.02	36	0.15				
Total lymphocytes < 1.1 × 10^9^/L	7.00	1.07	5	0.15				
Presence of risk factors for immunosuppression	7.05	1.59	27	0.23				
Immunomodulatory drugs					0.33^*^	0.94 ± 0.08	0.72 ± 0.32	0.83 ± 0.18
Recombinant interferon γ	5.59	1.50	5	0.27				
IgG	5.91	1.90	9	0.32				
Recombinant GM-CSF	6.23	1.63	9	0.26				
Tα1	7.32	1.84	27	0.25				
Immunomodulatory therapy required dynamic monitoring of immune function	8.41	0.80	55	0.09		0.92 ± 0.09	0.72 ± 0.34	0.82 ± 0.18
Endpoint of immunomodulatory therapy					0.01	0.91 ± 0.10	0.69 ± 0.28	0.80 ± 0.15
mHLA-DR ≥ 15,000 AB/C	6.91	1.44	14	0.21				
Percentage of mHLA-DR ≥ 60%	6.73	1.49	14	0.22				
Total lymphocytes ≥ 1.1 × 10^9^/L	6.95	1.05	0	0.15				

^*^P < 0.05. *BMI* body mass index, *GM-CSF* granulocyte–macrophage colony-stimulating factor, *Ig* immunoglobulin, *mHLA-DR* monocyte human leukocyte antigen DR, *Th* helper T cell, *Tα1* thymosin α1

**Fig. 2:**
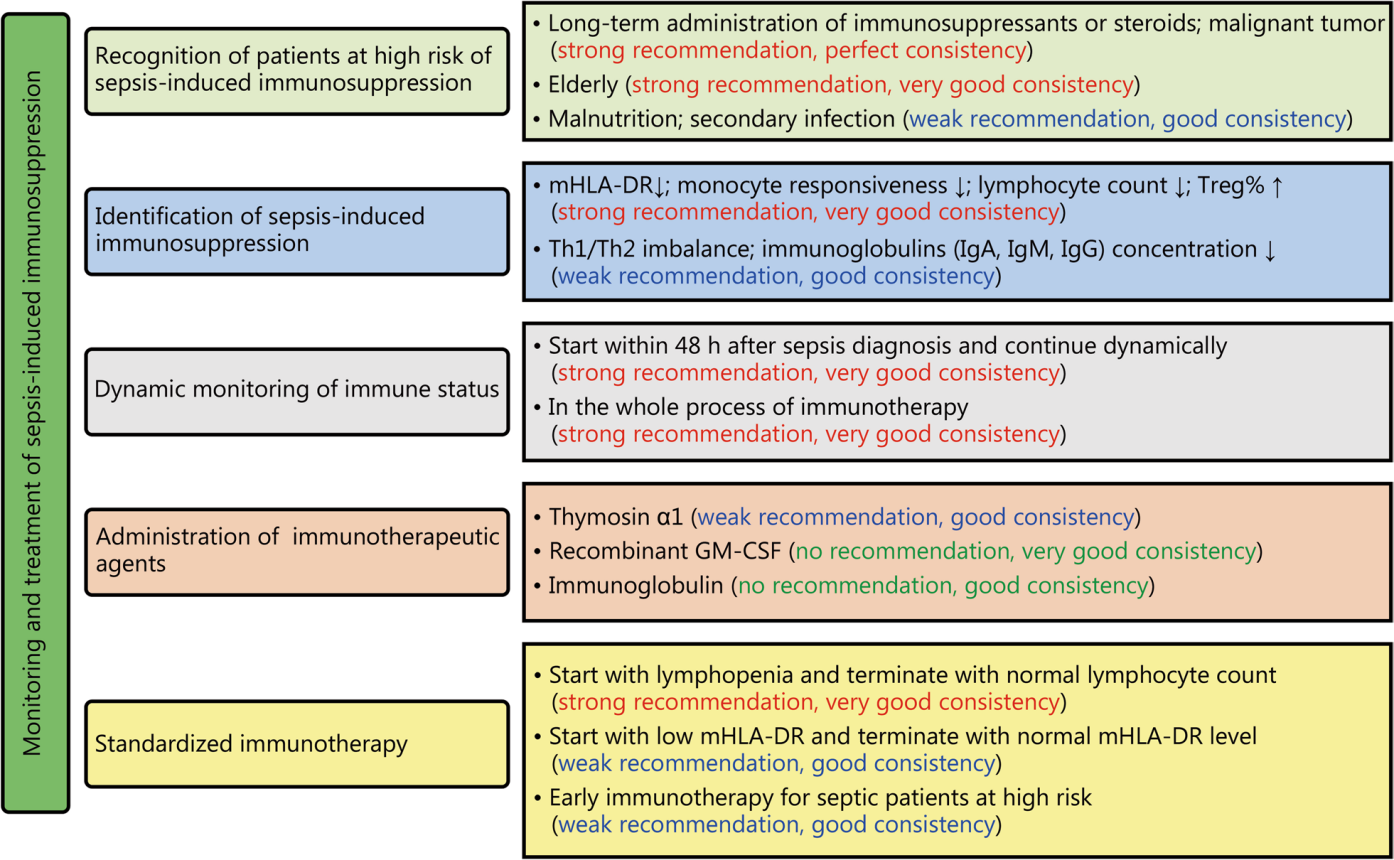
Summary of the recommendations on the monitoring and treatment of sepsis-induced immunosuppression. mHLA-DR monocyte human leukocyte antigen DR, GM-CSF granulocyte–macrophage colony-stimulating factor, Treg regulatory T cell

Based on the median and IQR of each item, the RAM is used to give consistency evaluation and recommendation for each item. In consistency evaluation, 2 items were rated as perfect consistency, 13 as very good consistency, and 9 as good consistency. In terms of recommendation, 14 items were rated as strong recommendation, 8 as weak recommendation, and 2 no recommendation (Table 6 and Fig. 2).

**Table 6:** Summary of consensus recommendations

Consensus	Consistency evaluation and recommendation
Patients with sepsis had immunosuppression	
Presence of innate immunosuppression	Very good consistency, strong recommendation
Presence of acquired immunosuppression	Very good consistency, strong recommendation
Sepsis-related immune function monitoring	
Initiated immune monitoring within 48 h after diagnosis of sepsis	Very good consistency, strong recommendation
Need for dynamic monitoring of immune status	Very good consistency, strong recommendation
Patients with sepsis had high risk factors for immunosuppression	
Elderly (age ≥ 65 years old)	Very good consistency, strong recommendation
Patients with malignant tumors under radiotherapy/chemotherapy within 3 months	Perfect consistency, strong recommendation
Patients undergoing long-term immunosuppressive or steroids therapy	Perfect consistency, strong recommendation
Malnutrition (BMI < 18.5 kg/m^2^)	Good consistency, weak recommendation
Secondary infection	Good consistency, weak recommendation
Monitoring indicators for immunosuppression in patients with sepsis	
Decreased mHLA-DR	Very good consistency, strong recommendation
Reduced responsiveness of monocytes to endotoxin stimulation	Very good consistency, strong recommendation
Decreased peripheral blood lymphocyte count	Very good consistency, strong recommendation
Increased regulatory T cell ratio	Very good consistency, strong recommendation
Th1/Th2 balance disorder	Good consistency, weak recommendation
Decreased peripheral blood immunoglobulin (IgA, IgM and IgG) concentrations	Good consistency, weak recommendation
Immunomodulatory therapy for sepsis	
mHLA-DR < 15,000 AB/C or percentage of mHLA-DR < 60%	Good consistency, weak recommendation
Total lymphocytes < 1.1 × 10^9^/L	Good consistency, weak recommendation
Presence of risk factors for immunosuppression	Good consistency, weak recommendation
Immunomodulatory drugs	
IgG	Good consistency, no recommendation
Recombinant GM-CSF	Very good consistency, no recommendation
Tα1	Good consistency, weak recommendation
Immunomodulatory therapy required dynamic monitoring of immune function	Very good consistency, strong recommendation
Endpoint of immunomodulatory therapy	
mHLA-DR ≥ 15,000 AB/C or percentage of mHLA-DR ≥ 60%	Good consistency, weak recommendation
Total lymphocytes ≥ 1.1 × 10^9^/L	Very good consistency, strong recommendation

*GM-CSF* granulocyte–macrophage colony -stimulating factor*, Ig* immunoglobulin, *mHLA-DR* monocyte human leukocyte antigen DR, *Th* helper T cell, *Tα1* thymosin α1

### Content of this consensus

#### Immunosuppression in patients with sepsis

*Evidence* The innate immune system is the body’s first line of defense against invasion by pathogens, which is manifested as a rapid and non-specific immune response. The innate immune response is mediated by a variety of innate immune cells including neutrophils, monocytes/macrophages, dendritic cells, and natural killer cells. Substantial evidence indicate that the innate immune response is significantly impaired in the early stage of sepsis, and its severity and duration are closely related to the clinical prognosis of septic patients [4, 16]. Innate immune function is impaired by several mechanisms, including dysfunction in neutrophil recruitment and migration [17–20], endotoxin tolerance in monocytes [21–24], reduced dendritic cell count and malfunction [25–29], and impaired natural killer cytotoxicity [30–32].

Compared with the innate immune response, the acquired immune system responds slowly, but can specifically initiate immune responses against different antigens and generate immune memory to deal with the second hit of the same antigen. Acquired immune responses include cellular immunity and humoral immunity, in which T and B lymphocytes perform the main functions, respectively. In the septic state, one of the hallmarks of impaired acquired immune response is lymphopenia, which is manifested by the absence of circulating T and B lymphocytes [33–39]. A previous study showed that persistent lymphopenia is significantly associated with increased incidence of nosocomial infection and mortality in patients with sepsis [40]. In terms of potentially involved mechanisms, enhanced apoptosis and dysfunction of acquired immune cells occur during sepsis, as evidenced by decreased number of T lymphocytes and abnormal distribution of T cell subsets [33, 41–43], and the latter included increased proportion of regulatory T cells (Tregs) with increased immunosuppressive activity [44–46], imbalance in helper T cell (Th)1/Th2 [47, 48], impaired B lymphocyte function, and low immunoglobulin levels [49, 50].

*Suggestions* (1) The expert panel recommended to identify innate immunosuppression in sepsis (strong recommendation, very good consistency). (2) The expert panel recommended to identify acquired immunosuppression in sepsis (strong recommendation, very good consistency).

#### Immune function monitoring during sepsis

*Evidence* Dynamic monitoring of immune function changes and early identification of immune dysfunction are prerequisites to effectively slow the progression of sepsis and improve the prognosis. However, the roles and characteristics of immune function status over time in the development and progression of sepsis remain unknown. Previous studies have shown that both the innate immunity and acquired immune cells undergo various degrees of functional changes within 48 h after the onset of sepsis, and the occurrence of immunosuppression during this period is closely related to a persistent multiple organ dysfunction and poor prognosis [51, 52]. Gouel-Chéron et al. [53] followed up the patients with trauma and found that changes in IL-6 and mHLA-DR levels at 1–2 d after admission were associated with the onset of sepsis. Indeed, a variety of immune monitoring indicators showed significant changes within 48 h after the occurrence of sepsis, including reduced mHLA-DR level and decreased lymphocyte count, which were significantly correlated with persistent immunosuppression and secondary infection [23, 51]. Therefore, monitoring the immune status within 48 h after the onset of sepsis provides important reference value for comprehensive understanding of the immune function status and dynamic assessment of disease progression in septic patients.

Dynamic monitoring of the host immune status can effectively notify the progression and severity of immunosuppression during sepsis [54–58]. When sepsis occurs, the functions and numbers of immune cells show dynamic alterations. In the early stage of sepsis, leukocytes in circulation are strongly activated, which leads to an effector state characterized by an excessive systemic inflammatory response. In the persistent state of sepsis, the body shows irreversible immunosuppression, or even immune paralysis, which induces secondary infection and moreover, opportunistic pathogen infection, a major factor affecting the long-term prognosis of patients [52, 59, 60]. Monneret et al. [23] found there was no significant difference in mHLA-DR levels between survivors and non-survivors in the early stage of sepsis; however, a significantly decreased mHLA-DR level in persistent sepsis was an independent risk factor for death. Venet et al. [61] conducted dynamic monitoring of immune status in critically ill coronavirus disease 2019 (COVID-19) patients, revealing significant differences in immune response-related indicators, such as lymphopenia, decreased mHLA-DR levels and inflammatory factor expression, between virus-infected survivors and non-survivors 3 weeks before intensive care unit (ICU) admission. Therefore, dynamic monitoring of immune function can not only reveal the immune status of patients in real time, but also provide a basis for accurate adjustment of immunomodulatory regimens.

*Suggestions* (1) The expert panel recommended to start immune monitoring within 48 h after sepsis diagnosis (strong recommendation, very good consistency). (2) The expert panel recommended to initiate dynamic monitoring of immune status in patients with sepsis (strong recommendation, very good consistency).

#### High risk factors for immunosuppression in patients with sepsis

*Evidence* Augus et al. [62] found that the incidence of sepsis was significantly higher in elderly patients, especially in those over 65 years, and increased rapidly with the age. A secondary analysis of the REGARDS cohort confirmed that elderly patients over 65 years had significantly higher odds of developing sepsis than middle-aged patients, which might be related to the comorbidity of multiple chronic diseases in elderly patients [63]. A prospective cohort study showed that the mortality rate of elderly patients with sepsis over 65 years was significantly higher than that of middle-aged patients, with immunosuppression particularly evident in the elderly patients who died in the study, manifesting as persistent lymphopenia, decreased functional T lymphocytes, increased Tregs and immunosuppressive T lymphocytes [40]. A post-hoc analysis of the ETASS study found that the incidence of sepsis-induced immunosuppression in elderly patients (≥ 60 years) was about twice that of non-elderly patients, and the mortality rate of elderly patients with sepsis-induced immunosuppression was significantly higher than that of elderly patients without immunosuppression [60]. In addition to age, nutritional status has also been confirmed to be closely related to the occurrence and development of sepsis. Nutritional status is often reflected by the body mass index (BMI), and malnutrition is a common issue for the hospitalized patients, usually manifested by underweight (BMI < 18.5 kg/m^2^). It is well established that the nutritional status of patients with sepsis is significantly associated with worse clinical outcomes and mortality [64]. A Chinese prospective cohort study confirmed that BMI is an independent risk factor for long-term mortality in patients with sepsis, and 90-day mortality was significantly higher in low-weight patients with sepsis than that in normal-weight and over-weight patients with sepsis [65]. A Japanese cohort study also demonstrated an association between malnutrition and increased 28-day mortality in patients with sepsis [66].

In addition to various malignancies, radiotherapy/chemotherapy required for malignant tumor treatment can cause various extend of immunosuppression, which increases the probability of infection in these patients [67]. Large-scale epidemiological studies have shown that patients with tumors are more likely to develop severe sepsis than patients without tumors [68, 69]. Further investigation confirmed that the mortality rate of septic patients with tumors was significantly higher than that of septic patients without tumors, and the duration of hospital stay was significantly prolonged as well. Additionally, patients with tumors undergoing radiotherapy/chemotherapy were more prone to neutropenic sepsis, especially patients with hematological malignancies [70, 71]. Meanwhile, long-term use of steroids and immunosuppressants has been shown to cause persistent immunocompromised states. In an analysis of the REGARDS cohort, Chaudhary et al. [72] found that chronic steroid users were twice as likely to develop sepsis as those not using steroids. Another study showed that severe sepsis cases with acquired immunodeficiency syndrome (AIDS) had significantly higher incidence of opportunistic infections and in-hospital mortality compared with those without AIDS [73].

Numerous data have suggested that sepsis-induced immunosuppression makes septic patients more susceptible to concurrent infections, which results in a further increase in late mortality. A prospective cohort study analyzed the incidence of secondary infections and its impact on prognosis in septic patients after ICU admission, showing that septic patients with secondary infections had prolonged hospital stay, significantly more complications, and higher mortality than those without secondary infections [74]. In addition, a transcriptome sequencing study found that the expression of immunosuppression-related genes in peripheral blood leukocytes of severe septic patients with secondary infections was significantly upregulated, and abnormal glucose metabolism was significantly enriched.

*Suggestions* (1) The expert panel recommended elderly (≥ 65 years) as a high-risk factor for immunosuppression in patients with sepsis (strong recommendation, very good consistency). (2) The expert panel recommended malignant tumor as a high-risk factor for immunosuppression in patients with sepsis (strong recommendation, perfect consistency). (3) The expert panel recommended long-term immunosuppressant or steroid therapy as a high-risk factor for immunosuppression in patients with sepsis (strong recommendation, perfect consistency). (4) The expert panel recommended malnutrition (BMI < 18.5 kg/m^2^) as a high-risk factor for immunosuppression in patients with sepsis (weak recommendation, good consistency). (5) The expert panel recommended secondary infection as a high-risk factor for immunosuppression in sepsis patients (weak recommendation, good consistency).

#### Monitoring indicators of immunosuppression in patients with sepsis

*Evidence* Decreased mHLA-DR levels have good discriminative capacity and clinical value in the assessment of disease severity and prognosis in patients with sepsis [75]. Previous studies have reported that mHLA-DR levels in the peripheral blood of patients with severe sepsis and septic shock are significantly lower than those of healthy individuals, and the non-survivor group showed persistently lower mHLA-DR expression than the survivor group [23, 76]. Drewry et al. [77] observed that mHLA-DR levels in non-survival patients were significantly lower than those of surviving patients on 1–8 d after the onset of sepsis, and were closely related to the occurrence of secondary infection. In addition, declined mHLA-DR levels only gradually returned to normal 6 months after recovery and discharged of patients with sepsis [78]. Decreased mHLA-DR expression was highly consistent with lymphopenia when assessing the disease severity and poor prognosis of sepsis patients [78]. Therefore, dynamic monitoring of mHLA-DR expression has an important guiding significance for evaluating disease progression, disease severity and prognosis among septic patients.

Decreased tumor necrosis factor-α (TNF-α) in monocytes stimulated with endotoxin/lipopolysaccharide (LPS) is one of the main features of impaired monocyte activation, which is closely related to immunosuppression in patients with sepsis. Albert Vega et al. [79] observed that after LPS stimulation of peripheral blood monocytes derived from patients with sepsis, TNF-α production was significantly lower than that detected in normal healthy controls. Ploder et al. [80] conducted a dynamic analysis of peripheral blood monocytes in patients with multiple trauma-induced sepsis and confirmed that compared with the survivor group, peripheral blood monocytes in the non-survivor group displayed persistently reduced TNF-α production with LPS stimulation, suggesting it might be one of the important indicators for prognosis evaluation in patients with sepsis. Hall et al. [81] observed in a multicenter cohort study that if LPS-induced TNF-α production in peripheral blood monocytes from patients with multiple organ dysfunction combined with immune paralysis was lower than 200 pg/ml, the incidences of infection aggravation and secondary infections were significantly increased. Therefore, reduced responsiveness of monocytes to endotoxin stimulation, as direct evidence for impaired monocyte activation, could effectively reflect the immunosuppressive state of septic patients.

Lymphopenia including decreased amounts of T and B lymphocytes in peripheral blood is one of the major manifestations of the dysregulated acquired immune responses. Daix et al. [82] conducted flow cytometry analysis of peripheral blood lymphocytes in 781 patients with sepsis, showing significant positive correlations between the decrease of CD3^+^ lymphocyte counts and sepsis severity and elevated mortality. Previous studies also reported that in the early stage of sepsis, enhanced apoptosis of CD4^+^ T lymphocytes and B lymphocytes occurred, and the absolute counts of peripheral blood lymphocytes decreased sharply, with persistent lymphopenia directly related to increased mortality in septic patients [35, 57]. Vulliamy et al. [57] showed that compared with the survival group, the number of peripheral blood lymphocytes continued to decrease 2–7 d after the onset of sepsis in the non-survivor group, with the risk of death increasing by 3.5 folds. Therefore, dynamic observation of changes in the number of lymphocytes can not only reveal the state of the acquired immune response, but also help evaluate the balance between innate immunity and acquired immunity.

As important immune regulatory cells, Tregs participate in the process of sepsis-related immune paralysis by promoting T lymphocyte apoptosis, inhibiting T lymphocyte proliferation, and promoting an anti-inflammatory state. Previous studies have found that the increased proportion of Tregs in the peripheral blood of patients complicated with severe burns or trauma is closely related to the occurrence and progression of sepsis, and a persistently elevated level of Tregs is considered as an important factor for increased mortality in patients with sepsis [46, 83]. In addition, some data have confirmed that increased proportion of Tregs in peripheral blood can occur in the early stage of sepsis and cause an imbalance of the Th subset termed Th17, whose change is closely associated with multiple organ dysfunction and poor prognosis [84, 85].

Studies have also shown that an imbalanced Th1/Th2 ratio is one of the main manifestations of impairment in T lymphocyte differentiation, which correlates closely with abnormal production of cytokines and impaired T lymphocyte effectors. Several clinical trials have shown that T lymphocytes in the peripheral blood isolated from patients with sepsis and septic shock appeared to polarize towards Th2, which resulted in imbalanced Th1/Th2 ratio. This is one of the most important causes of immunosuppression, and is significantly related to high mortality [47, 48]. In a single-center prospective observational cohort study, Xue et al. [86] found that Th1/Th2 ratios in peripheral blood from septic patients admitted to ICU were significantly lower than those of patients without sepsis. They also found that, compared with the survivor group, Th1/Th2 ratios in peripheral blood from the non-survivor group continued to decrease 0–7 d after the onset of sepsis, which was significantly associated with increased incidence of ICU-related infections and mortality. Currently, due to the limitation in laboratory platform and technical requirements, and Th1/Th2 ratio detection is not widely used in clinical immune assessment; nevertheless, monitoring of Th1/Th2 balance has certain reference value for understanding the immune status.

Decreased immunoglobulin (IgA, IgM and IgG) amount is one of the potential manifestations of immunosuppression, whose value in the assessment of immune impairment in patients with sepsis remains controversial. Shankar-Hari et al. [87] conducted a systematic review and meta-analysis, and found that circulating IgG levels in patients with sepsis were significantly decreased, but not significantly associated with sepsis severity and mortality. Průcha et al. [88] analyzed 1513 samples from 708 patients, and revealed that significantly increased mortality in severe sepsis cases was parallel with low IgG levels, while patients with septic shock had lower IgG and IgM levels, showing significantly higher mortality than patients with normal IgG and IgM levels. In addition, the combined use of reduced peripheral blood IgA, IgM and IgG levels could significantly improve the early determination of prognosis in patients with sepsis and septic shock [89]. Recently, Alagna et al. [90] conducted a multicenter randomized controlled study of 956 patients with severe sepsis and septic shock, and found that high IgA and IgG levels on the day of diagnosis of sepsis were significantly associated with decreased 90-day survival in patients with sepsis, whereas changes in IgM levels were not significantly associated with the survival. Therefore, immunoglobulin regulation is an important immune effector mechanism in the host, and the combined detection of multiple indicators may help understand the abnormal immune response in sepsis. However, its exact clinical guiding significance needs further investigation by large-sample multicenter clinical trials.

*Suggestions* (1) The expert panel recommended decreased mHLA-DR as a monitoring indicator of immunosuppression in patients with sepsis (strong recommendation, very good consistency). (2) The expert panel recommended decreased monocyte responsiveness to endotoxin stimulation as a monitoring indicator of immunosuppression in patients with sepsis (strong recommendation, very good consistency). (3) The expert panel recommended reduced lymphocyte count as a monitoring indicator of immunosuppression in patients with sepsis (strong recommendation, very good consistency). (4) The expert panel recommended increased proportion of Tregs as a monitoring indicator of immunosuppression in patients with sepsis (strong recommendation, very good consistency). (5) The expert panel recommended to include Th1/Th2 ratio imbalance as a monitoring indicator of sepsis-induced immunosuppression (weak recommendation, good consistency). (6) The expert panel recommended to include decreased concentrations of immunoglobulins (IgA, IgM and IgG) as a monitoring indicator of sepsis-induced immunosuppression (weak recommendation, good consistency).

#### Starting point for immunomodulatory therapy of sepsis

*Evidence* Emerged evidence has demonstrated that immunosuppression can occur at any time after the onset of sepsis [11, 16, 91]. It is inappropriate to blindly conduct immunomodulatory therapy in patients with sepsis, and it is extremely important to identify the starting point for immunomodulatory therapy. Therefore, we explored the starting point for immunomodulatory therapy in patients with sepsis based on completed clinical trials and the clinical practice experiences of ICU specialists. Age is one of the important factors affecting immune function [92, 93]. Current studies have found that lymphocyte count and mHLA-DR levels in the elderly patients with sepsis are significantly lower than those of the younger patients, and elderly can rapidly develop sepsis-induced immunosuppression within 48 h of the onset of sepsis [40, 60]. Patients with malignant tumors undergoing radiotherapy or chemotherapy and those undergoing treatment with long-term immunosuppressants and steroids often suffer from sepsis due to immunosuppression [94–96]. Reversal of immunosuppression helps clear pathogenic bacteria, and it is recommended to consider immunomodulatory therapy as soon as possible after discussion with specialists.

Lymphocyte count and mHLA-DR levels are two commonly used clinical indicators for immune monitoring, which have been used to select immunosuppressed patients for immunomodulatory therapy [97–101]. In a study conducted by Cheng et al. [99], COVID-19 patients with lymphocyte count below 800/µl were selected for recombinant granulocyte–macrophage colony-stimulating factor (GM-CSF) treatment. Francois et al. [97] selected septic shock patients with lymphocyte count below 900/µl for IL-7 therapy. Meisel et al. [98] selected septic patients with mHLA-DR under 8000 AB/C for recombinant GM-CSF treatment by consecutive monitoring for 2 d. These studies suggested that decreased lymphocyte count and reduced mHLA-DR expression could be used as biomarkers for initiating immunomodulatory therapy.

*Suggestions* (1) The expert panel recommended to consider immunomodulatory therapy for septic patients with decreased peripheral blood lymphocyte count (absolute count < 1.1 × 10^9^/L) (strong recommendation, very good consistency). (2) The expert panel recommended to consider immunomodulatory therapy for septic patients with decreased mHLA-DR expression (percentage < 60% or absolute count < 15,000 AB/C) (weak recommendation, good consistency). (3) The expert panel recommended to consider immunomodulatory therapy for septic patients with high risk factors for immunosuppression (elderly, malignant tumor, long-term use of immunosuppressive drugs, etc.) (weak recommendation, good consistency).

#### Immunomodulatory drugs for sepsis

*Evidence* Thymosin α1 (Tα1) plays an important immunomodulatory role in both the innate and adaptive immune systems [102]. Several clinical trials using Tα1 to treat sepsis have been conducted [103, 104]. As early as 2007, Lin [105] conducted the first multicenter RCT that used Tα1 plus ulinastatin to treat sepsis in China. The trial was divided into two phases according to the different doses: 91 patients with sepsis were enrolled in the first phase, and the treatment group (44/91) received ulinastatin 100,000 U, 3 times per day and 1.6 mg Tα1 once daily; however, the preliminary results showed that the 28-day mortality in patients with sepsis had no significant difference between the treatment and control groups. Having considered the result might be related to insufficient therapeutic doses, the second phase of the study doubled the doses of the therapeutic drugs and enrolled 342 patients with sepsis, and the treatment significantly reduced 28-day mortality (*P* = 0.0088) and 90-day mortality (*P* = 0.0054) in patients with sepsis compared with the control group. Meanwhile, mHLA-DR expression in the treatment group increased significantly. Wu et al. [106] conducted a multicenter RCT using Tα1 to treat sepsis (the ETASS study), which showed that Tα1 treatment could reduce 28-day all-cause mortality in patients with sepsis. A meta-analysis included 19 studies showed that Tα1 treatment significantly improved the clinical prognosis of patients with sepsis, but the sample size included in the analysis was small [107]. Currently, a multicenter, randomized, double-blind, placebo-controlled clinical trial using Tα1 to treat sepsis (the TESTS study, NCT02867267) has been completed [108]. Recent studies have found that Tα1 is also effective in the treatment of patients with COVID-19. In a retrospective study including 76 patients with severe COVID-19, Tα1 treatment significantly reduced patient’s mortality (*P* < 0.05) and increased CD4^+^ and CD8^+^ T cell counts compared with the control group [109]. Another multicenter retrospective cohort trial including 334 patients with COVID-19 in 8 hospitals in China [110] found that Tα1 treatment significantly prolonged the 28-day survival rate of critically ill COVID-19 patients. However, a retrospective study including 275 patients with COVID-19 found no beneficial effects for Tα1 on the recovery of CD4^+^ and CD8^+^ T cell counts and viral clearance during COVID-19 convalescence [111]. Another study including 771 critically ill COVID-19 patients also demonstrated that Tα1 did not reduce the mortality in critically ill COVID-19 patients [112].

Immunoglobulin is a natural protein secreted by B cells. The SBITS study [113] (*n* = 624) and the ESSICS study [114] (*n* = 218) are two RCTs with currently the largest sample sizes using intravenous immunoglobulin (IVIG) for sepsis treatment. In the SBITS trial, patients in the IVIG group (321/624) were given 0.6 g/kg IgG infusion immediately after enrollment and 0.3 g/kg IgG on day 1. No significant difference in the 28-day mortality was found between the IVIG group and the control group. A total of 218 postoperative cardiac surgery patients with severe inflammatory reactions were included in the ESSICS study in which the control group received 0.1% albumin, while the IVIG-treated group received the same volume of 10% IgG. There was no significant difference in the 28-day mortality between the two groups (31.5% in the control group vs. 39.1% in the IVIG-treated group). These two large RCTs suggest that IVIG does not reduce mortality in patients with sepsis. Subsequently, Iizuka et al. [115] designed a large retrospective paired study and again demonstrated that the use of IVIG therapy did not improve the prognosis of patients with sepsis. The INSTINCT study [116] found that IVIG (25 g/d for 3 d) did not reduce the 180-day mortality in patients with skin and soft tissue infections. Nakamura et al. [117] conducted a study with a small sample size and observed that compared with continuous IVIG treatment for 3 d (5 g/d), a single infusion of IVIG (15 g/d) on the first day shortened the duration of ICU stay. In 2013, Alejandria et al. [118] conducted a meta-analysis in which 10 studies of IVIG treatment for sepsis (*n* = 1430) showed that the 28–180-day mortality rates were 29.6% in the IVIG group and 36.5% in the placebo group (*RR* = 0.81; 95% CI 0.70–0.93), whereas other 7 studies using IgM-enriched IVIG (IVIGM) (*n* = 528) showed that the 28–60-day mortality rates were 24.7% in the IVIGM group and 37.5% in the placebo group (*RR* = 0.66; 95% CI 0.51–0.85); however, it should be noted that both meta-analyses of IVIG and IVIGM treatment had moderate-to-high risk of bias. Recently, Laupland et al. [119] conducted a meta-analysis that included high-quality studies using IVIG to treat sepsis, confirming that IVIG did not improve the prognosis of patients with sepsis (*OR* = 0.96; 95% CI 0.71–1.3). When studies with high risk of bias were excluded, the effect of IVIG treatment might no longer be manifested. Consequently, the “surviving sepsis campaign” guideline did not recommend IVIG for the treatment of sepsis [120]. Nevertheless, further studies are needed for IVIGAM treatment to demonstrate its efficacy. Hentrich et al. [121] used intravenous IgM- and IgA-enriched IVIG (IVIGMA) to treat sepsis patients with neutropenia from chemotherapy, and found that IVIGMA treatment did not improve the prognosis of those patients. Welte et al. [122] used IVIGMA to treat patients with severe community-acquired pneumonia, and showed that IVIGMA did not statistically increase ventilator-free days significantly; however, subgroup analysis found that IVIGMA reduced mortality in patients with elevated C-reactive protein (≥ 70 mg/L) and low IgM (≤ 0.8 g/L).

Recombinant GM-CSF is a growth factor that stimulates the proliferation and differentiation of various immune cells. Orozco et al. [123] used GM-CSF [3 μg/(kg·d)] to treat septic patients with nontraumatic abdominal infections, revealing that GM-CSF reduced the duration of antibiotic use and hospital stay, and decreased infection-related complications, but did not reduce the in-hospital mortality in patients with sepsis. In 2009, Meisel et al. [98] used GM-CSF [4 μg/(kg·d)] to treat septic patients with immunosuppression (mHLA-DR < 8000 AB/C for two consecutive days) and observed that mHLA-DR expression was significantly increased in all subjects of the treatment group compared with only in 15.8% patients of the control group. In 2011, Bo et al. [124] conducted a meta-analysis that included 2380 septic patients administered with G-CSF or GM-CSF and found that the treatment did not improve the prognosis of these patients, but further subgroup analysis found that GM-CSF treatment was beneficial to the clearance of pathogenic bacteria. In 2018, Pinder et al. [125] conducted a clinical observation, and showed that neutrophil phagocytosis in the 10 (100%) of the 10 patients in the GM-CSF group [3 μg/(kg·d) for 4 consecutive days] increased by more than 50%, while only 7 (44%) of the 16 patients in the control group reached the same level, demonstrating that GM-CSF is beneficial to the improvement of neutrophil phagocytosis. Currently, a large, multicenter, randomized, double-blind, placebo-controlled clinical trial (NCT02361528) using GM-CSF to treat septic patients with immunosuppression has been initiated, with the 28-day mortality and/or ICU-acquired infection rate as the primary endpoints [126].

*Suggestions* (1) The expert panel recommended to use Tα1 to treat septic patients with immunosuppression (weak recommendation, good consistency). (2) The expert panel did not recommended immunoglobulin for immunomodulatory therapy in septic patients with immunosuppression (no recommendation, good consistency). (3) The expert panel did not recommend recombinant GM-CSF for immunomodulatory therapy in septic patients with immunosuppression, but the symptomatic treatment can be considered for sepsis patients with leukopenia (no recommendation, very good consistency).

#### Immunomodulatory therapy requires dynamic monitoring of immune function

*Evidence* Sepsis is a complex disease with varying immune function among individuals, as well as substantially individual-dependent response to immunomodulatory therapy. Therefore, dynamic monitoring of immune function in the process of immunomodulatory therapy is helpful to understand the changes of immune function in real time. Previous studies have shown that dynamic observation of mHLA-DR and lymphocyte count could better assess the immune status and predict the prognosis of patients with sepsis [23, 76, 127–129]. Currently, mHLA-DR and lymphocyte count have been used for dynamic monitoring of immune function in immunomodulatory therapies. Meisel et al. [98] conducted a randomized, double-blind, controlled clinical trial that used GM-CSF to treat sepsis, as the first study to combine immune monitoring with immunomodulatory therapy. This study not only identified septic patients with immunosuppression by monitoring mHLA-DR for two consecutive days, but also set mHLA-DR ≥ 15,000 AB/C as the treatment endpoint for GM-CSF administration. A recent phase II clinical trial enrolled a total of 27 septic shock patients with severe lymphopenia and these patients were treated with recombinant IL-7 to treat sepsis; dynamic monitoring of lymphocyte count and lymphocyte subpopulations during IL-7 treatment found that absolute lymphocyte counts as well as circulating CD4^+^ and CD8^+^ T cell counts increased by more than threefold over baseline after IL-7 treatment [97]. Subsequently, in an ongoing phase II clinical trial including patients with sepsis administered with IL-7 treatment (NCT03821038, IRIS-7-C&D study), “lymphocyte count increased by more than 50%” was set as the primary endpoint [130]. These two studies suggested that immunosuppression in sepsis needs targeted immunomodulatory therapy, and mHLA-DR and lymphocyte count could be used as indicators for dynamic monitoring of immune function during sepsis.

*Suggestions* (1) The expert panel recommended that patients with sepsis undergo dynamic monitoring of immune function during immunomodulatory therapy (strong recommendation, very good consistency). (2) The expert panel recommended to monitor peripheral blood lymphocyte count to determine the endpoint of immunomodulatory therapy for sepsis (strong recommendation, very good consistency). (3) The expert panel recommended to monitor mHLA-DR to determine the endpoint of immunomodulatory therapy for sepsis (weak recommendation, good consistency).

## Data Availability

The datasets used and/or analyzed during the current study are available from the corresponding author on reasonable request.

## Abbreviations

WOS: Web of Science
CNKI: China National Knowledge Infrastructure
mHLA-DR: Monocyte human leukocyte antigen DR
RCTs: Randomized controlled trials
RAM: RAND appropriateness method
IQR: Interquartile range
Tregs: Regulatory T cells
Th: Helper T cell
IgG: Immunoglobulin
TNF-α: Tumor necrosis factor-α
LPS: Lipopolysaccharide
ICU: Intensive care unit
BMI: Body mass index
AIDS: Acquired immunodeficiency syndrome
COVID-19: Coronavirus disease 2019
GM-CSF: Granulocyte–macrophage colony-stimulating factor
Tα1: Thymosin α1
IVIG: Intravenous immunoglobulin
IVIGM: IgM-enriched IVIG
IVIGMA: IgM- and IgA-enriched IVIG
